# Supplementary material for: Novel Chemicals Derived from Tadalafil Exhibit PRMT5 Inhibition and Promising Activities against Breast Cancer
Source: Int J Mol Sci. 2022 Apr 27;23(9):4806. doi: 10.3390/ijms23094806 (PMC9103191; doi:10.3390/ijms23094806)
Supplement: Supplementary file 1 [file ijms-23-04806-s001.zip › ijms-1624470-supplementary.pdf]

Supplementary Materials

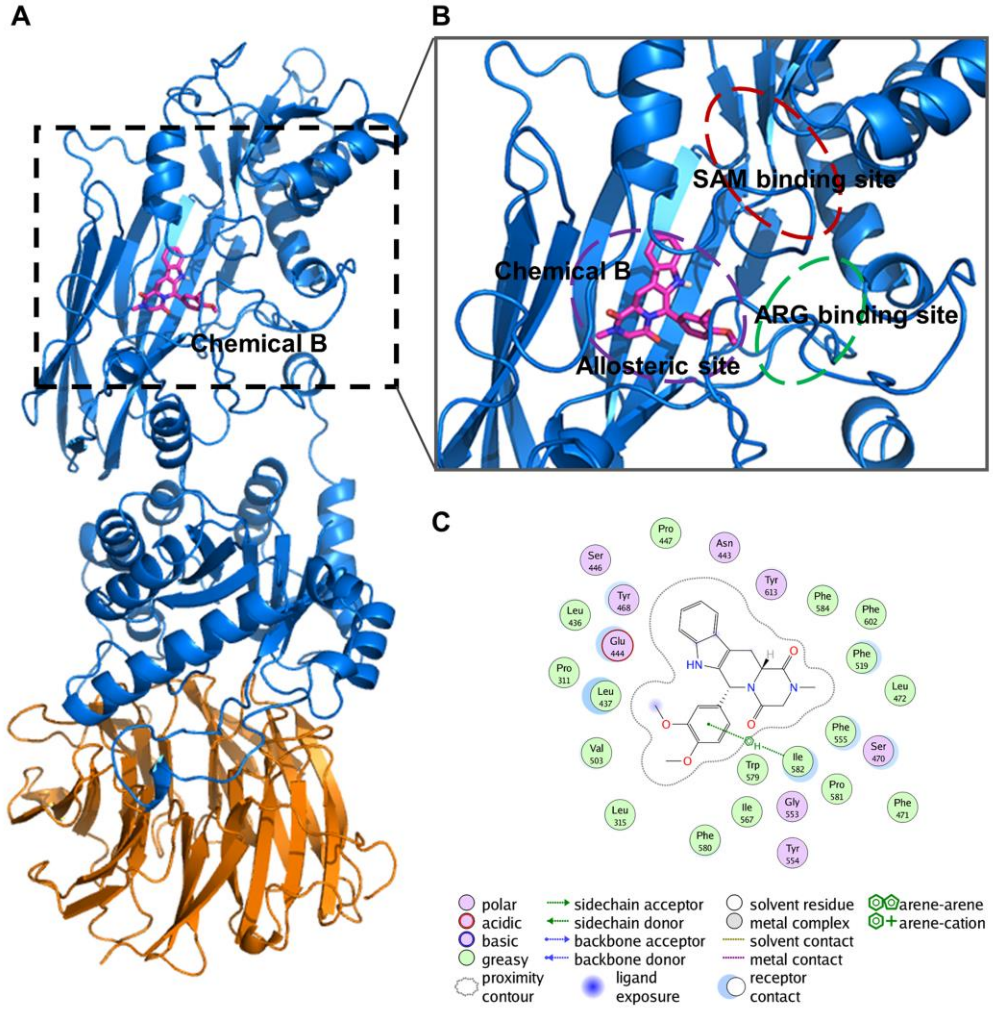

Figure S1. Binding interactions of chemical B with human PRMT5. **(A)** Overall structure of PRMT5:MEP50 complexed with chemical B at an allosteric site. PRMT5 is shown as a blue ribbon, MEP50 is shown as an orange ribbon, and chemical B is shown as purple sticks. **(B,C)** Detailed binding interactions of chemical B with human PRMT5.

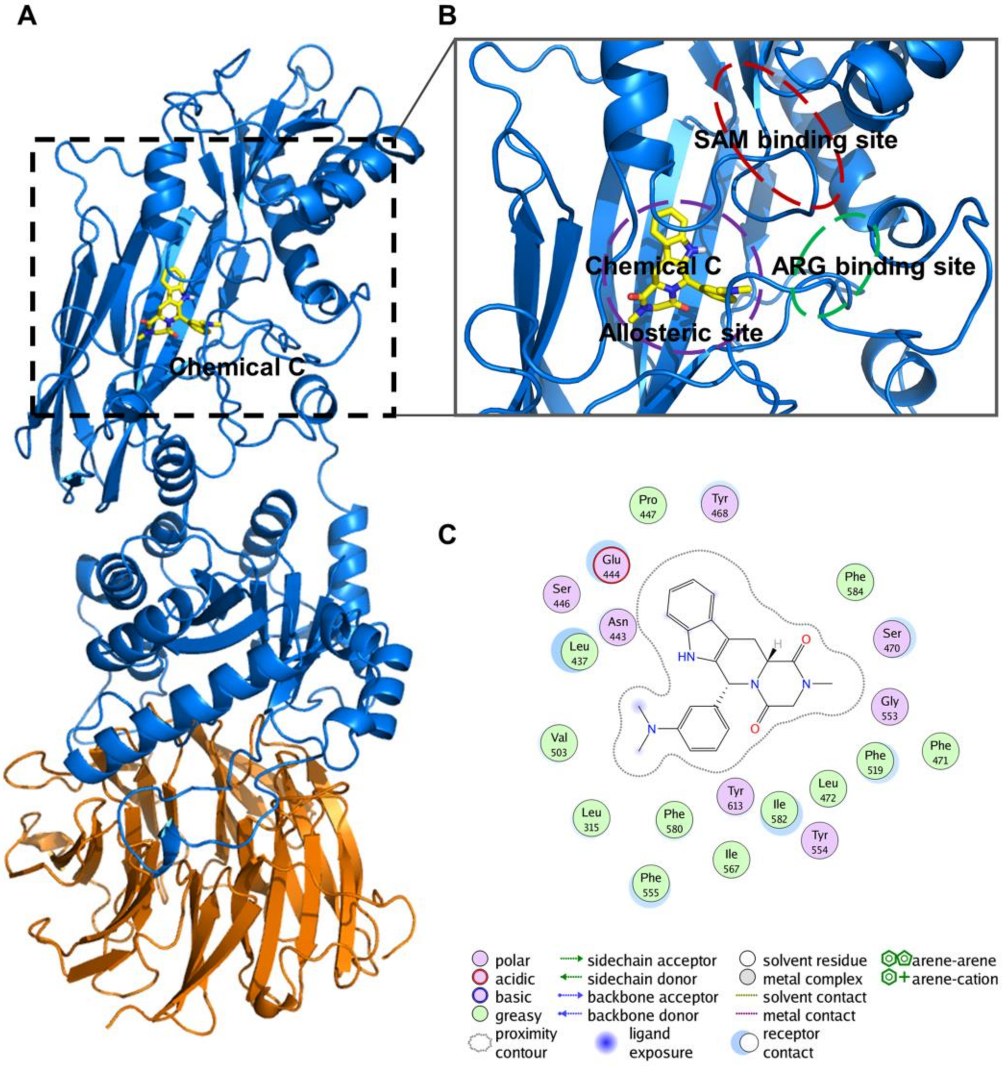

Figure S2. Binding interactions of chemical C with human PRMT5. **(A)** Overall structure of PRMT5:MEP50 complexed with chemical C at an allosteric site. PRMT5 is shown as a blue ribbon, MEP50 is shown as an orange ribbon, and chemical C is shown as yellow sticks. **(B,C)** Detailed binding interactions of chemical C with human PRMT5.

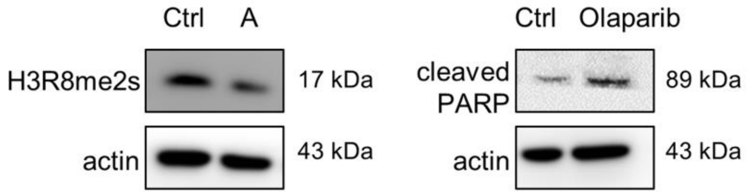

Figure S3. Target validation markers were detected in subcutaneous xenograft tumor models. Tumor tissues in Figure 12 were subjected to western blotting with anti-H3R8me2s and anti-cleaved PARP antibodies.
